# Food "Preservatives."

**Published:** 1899-12-02

**Authors:** 


### The Hospital

A Journal of

The Medical Sciences and Hospital Administration.

Vol. XXVII.—No. 688. EDITED BY SIR HENRY BURDETT, K.C.B., AND SOLOMON C. SMITH, M.D., M.R.C.P. [December 2, 1899.

### Food "Preservatives."

The readers of the daily papers, or at least those among them whose interest can be sustained beyond the columns of more or less apochryphal war news, can hardly have failed to notice the occasional appearance of a paragraph setting forth that a Departmental Committee on the use of "preservatives" in foods has held another meeting, and has received the evidence of one or more experts, usually in hearty and complete disagreement with each other, or, possibly, only with those who preceded them at the last recorded meeting. Not very long ago, for example, the Committee was told by Mr. Bond, the distinguished surgeon whose name is so well known in connection with many criminal investigations, that boric acid is practically harmless, and that he himself had taken it experimentally, in large and frequent doses, without any appreciable effect upon his health or comfort. On the 23rd instant there was the evidence of Dr. Handford, the Medical Officer of Health to the County of Notts, to the effect that he had of late years been considering the internal use of the same agent, and that in a large number of cases in which he had administered it as a remedy for diseased conditions of the urinary bladder he had been compelled to suspend its employment, on account of the irritation of the stomach and the loss of appetite which it occasioned. Some persons could take the usual dose of thirty grains of boric acid for weeks without distress, while in others it produced pain or discomfort within a few days. He related the case of an infant seven weeks old, suffering from diarrhœa, and who had been fed with milk containing seven grains of boric acid to the quart, which he described as "a large dose" for an infant, and one likely soon to produce injurious effects. On the same day Dr. Williams, the medical officer for Glamorgan, who gave evidence on behalf of the Incorporated Society of Medical Officers of Health, told the Committee that our knowledge of the physiological action of borax and of boric acid is so imperfect as to render it difficult to say what amount of these substances could be added (to food) with safety to the consumer. They should not be sanctioned as preservatives of milk and butter unless under stringent rules as to the quantities to be used; and in the present state of knowledge they should be prohibited altogether. "There was no doubt that the practice of drugging the public promiscuously and without their knowledge by incompetent people was highly dangerous." In his opinion we feel sure that all members of the medical profession will concur, and that they would welcome such a report from the Committee as would practically compel the Government to give the necessary attention to the subject, and to procure the enactment of laws for the protection of the community.

There is, of course, what is called the "commercial" aspect of the question, as well as the sanitary aspect; and we shall probably be told that the use of the so-called preservatives, by diminishing the perishability of the provision dealer's stock in trade, has an important influence in cheapening the food of the poor. Solicitude for the poor was professed by a notorious character, more than eighteen hundred years ago, and was then declared, on high authority, to be a mere cloak for other considerations. We have no confidence in the philanthropy of commerce. Commerce may not be immoral; but, as Huxley said of the government of the universe, it certainly appears to us to be "non-moral," and to hold that the "custom of the trade," taken together with the maxim *Caveat Emptor*, affords a sufficient justification for practices which most buyers, if they were made acquainted with them, would probably feel inclined to condemn. Moreover, in relation to the general question of food, it must be borne in mind that the object of food is to afford nourishment, not merely to be purchased and swallowed; and that any admixture which diminishes its nutritive properties may diminish its real value in even greater proportion than its price. We are strongly of opinion that much of the alleged necessity for preservatives arises entirely from slovenly and careless methods of preparation, and from avoidable delay in placing the articles upon the market; and that, if stringent laws were enacted upon the subject, the various trades concerned would speedily find methods of accomplishing what they would now doubtless declare to be impossible. Dr. Handford, to whom we have referred above, told the Committee that the addition of boric acid to fish tended rather to conceal decomposition than to prevent it; and, if this be so, it is obvious that its employment amounts to a highly injurious fraud upon the consumer. Moreover, if such additions are to be permitted at all, they will speedily lead to a new form of an old question. Who is to guarantee the purity and genuineness of the "preservatives"? What is "boric acid"? The new British Pharmacopœia tells us that "it should yield no *characteristic* reaction with the tests for lead or copper, and only the slightest reactions with the tests for iron, calcium, magnesium, potassium, sodium, ammonium, chlorides, and sulphates." The list suggests a very pretty witches' cauldron of possible or probable impurities, and renders it evident that the "preservative" itself should be as closely looked after as the foodstuffs which it is used to preserve.

